# Statistical distortion of supervised learning predictions in optical microscopy induced by image compression

**DOI:** 10.1038/s41598-022-07445-4

**Published:** 2022-03-02

**Authors:** Enrico Pomarico, Cédric Schmidt, Florian Chays, David Nguyen, Arielle Planchette, Audrey Tissot, Adrien Roux, Stéphane Pagès, Laura Batti, Christoph Clausen, Theo Lasser, Aleksandra Radenovic, Bruno Sanguinetti, Jérôme Extermann

**Affiliations:** 1 HEPIA, HES-SO, University of Applied Sciences and Arts Western Switzerland, Rue de la Prairie 4, 1202 Geneva, Switzerland; 2 Laboratory of Nanoscale Biology, School of Engineering, École Polytechnique Fédérale de Lausanne, 1015 Lausanne, Switzerland; 3 Wyss Center for Bio- and Neuroengineering, Geneva, Switzerland; 4 Department of Basic Neurosciences, Geneva Neuroscience Center, Faculty of Medicine, University of Geneva, Geneva, Switzerland; 5 Dotphoton SA, Zeughausgasse 17, 6300 Zug, Switzerland; 6 Max-Planck Institute for Polymer Research, Ackermannweg 10, 55128 Mainz, Germany

**Keywords:** Machine learning, Light-sheet microscopy, Phase-contrast microscopy, Imaging and sensing, Image processing

## Abstract

The growth of data throughput in optical microscopy has triggered the extensive use of supervised learning (SL) models on compressed datasets for automated analysis. Investigating the effects of image compression on SL predictions is therefore pivotal to assess their reliability, especially for clinical use. We quantify the statistical distortions induced by compression through the comparison of predictions on compressed data to the raw predictive uncertainty, numerically estimated from the raw noise statistics measured via sensor calibration. Predictions on cell segmentation parameters are altered by up to 15% and more than 10 standard deviations after 16-to-8 bits pixel depth reduction and 10:1 JPEG compression. JPEG formats with higher compression ratios show significantly larger distortions. Interestingly, a recent metrologically accurate algorithm, offering up to 10:1 compression ratio, provides a prediction spread equivalent to that stemming from raw noise. The method described here allows to set a lower bound to the predictive uncertainty of a SL task and can be generalized to determine the statistical distortions originated from a variety of processing pipelines in AI-assisted fields.

## Introduction

In the last years, an ever-growing community of optical microscopists is facing a massive data throughput, long-term storage costs, data transfer limitations and, more importantly, the need for automated quantitative data analysis, which has paved the way for extensive use of artificial intelligence (AI) methods. Supervised learning (SL) algorithms are routinely adopted to automate classification, segmentation, and artificial labelling of cellular or sub-cellular structures^1–3^, biological tissues^4–6^, as well as material defects^7–9^. SL approaches have reported remarkable results in various fields, such as medical screening^10,11^, single molecule localization^12,13^ and drug discovery^14,15^. Deep-learning (DL) algorithms have also been successfully employed for micrograph restoration, in particular for de-noising and spatial resolution enhancement^16–18^.

However, to deal with large training datasets and computational power constraints, SL models are ubiquitously executed on compressed imaging datasets. Despite producing visually faithful images, lossy compression algorithms are known to remove an unpredictable amount of information from the raw image. Moreover, compressed data often undergo additional processing before being used to train or test a SL model. Therefore, image compression can modify SL predictions with respect to when raw datasets are used and lead to unreliable scientific outcomes, based on how much the statistical distribution of the final predictions is altered. For this reason, the statistical distortions induced by compression need to be quantified to investigate the tolerability of image compression methods for SL applications.

To this end, it is crucial to measure the statistical distribution of the SL outcomes in the absence of compression, in other terms the prediction uncertainty associated to raw data. According to Begoli et al.^19^, the lifecycle of an AI process from the physical sample to the AI-assisted decisions is affected by multiple sources of uncertainty. Understanding how image compression affects the statistical distribution of SL outcomes can be ascribed to investigating the representational uncertainty of the AI pipeline, consisting in errors due to the data representation adopted for training or testing the SL model.

Here, we propose a method for quantifying the statistical distortions induced by compression on SL predictions. We first determine the predictive uncertainty of a trained SL model from the statistical noise of raw imaging data. Raw noise is measured via sensor calibration. As raw noise is unavoidable, our approach allows one to estimate the minimal level of representational uncertainty in SL predictions. Then, we compare outcomes obtained on compressed datasets to the raw predictive uncertainty by using a specific figure of merit, that will indicate the level of alteration of the SL outcomes statistics.

We implement this method to investigate the impact of image compression on the outcomes of cell segmentation tasks. To this end, we will consider three types of operations aimed at reducing data volume: pixel depth reduction, JPEG compression, as well as a metrologically accurate compression technique developed by the Dotphoton (DP) company (www.dotphoton.com). The DP method reports compression ratios from 5:1 to 10:1 after an initial image preparation step, in which image noise is replaced with a pseudo-random noise that closely mimics the statistical distribution of the raw pixel values. Although the noise replacement reduces the signal-to-noise ratio of each pixel by 1.2 dB, it allows to achieve high compression factors, as the pseudo-noise can be computed and makes the subsequent application of a standard lossless compression algorithm more efficient^20^.

## Results

### Raw data statistical noise

**Figure 1:**
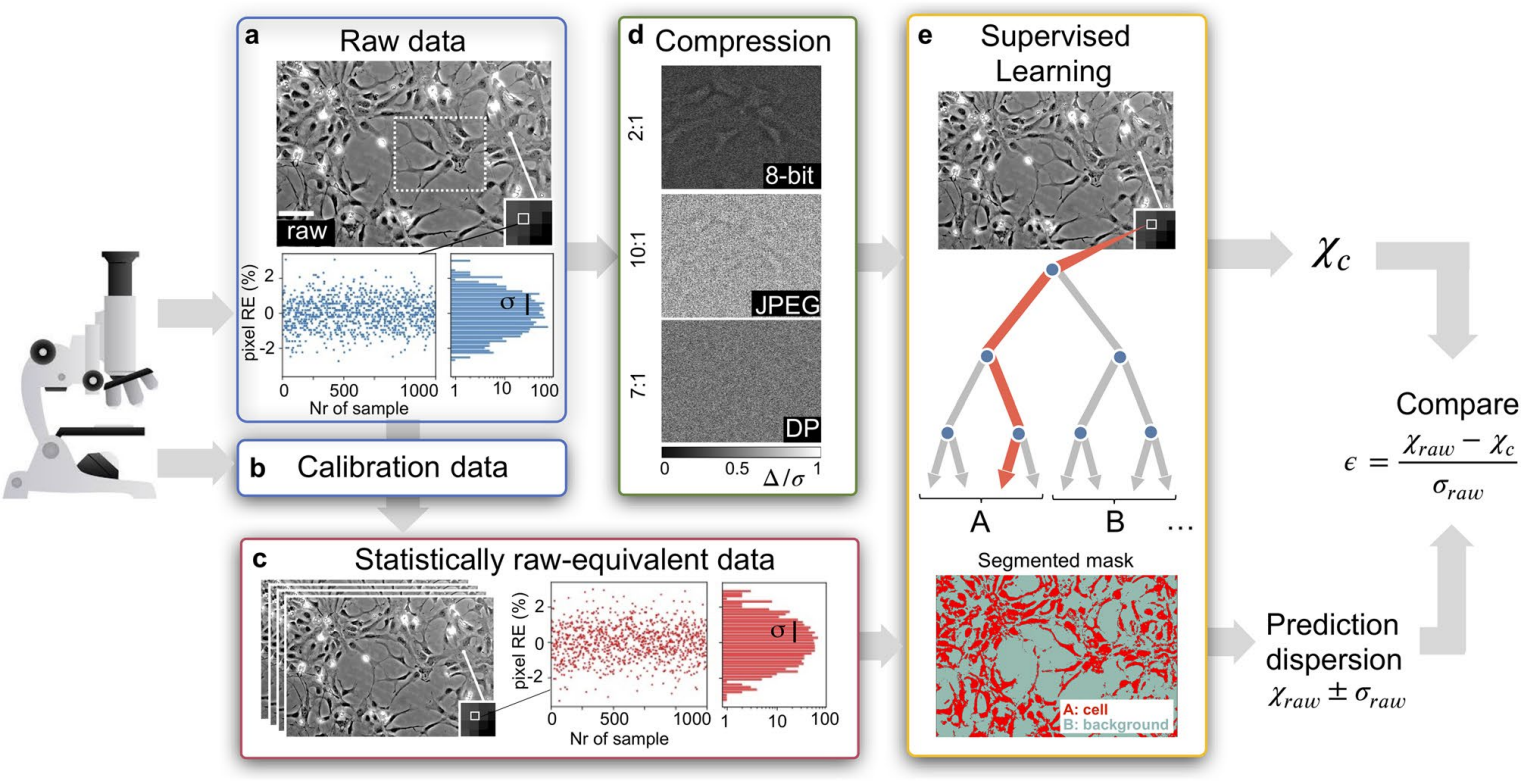
Quantification of the statistical distortions induced by image compression on SL predictions. (**a**) Raw imaging data from a microscope are intrinsically affected by noise: pixel values in a raw micrograph (in the figure a PC image of human neural stem cells (scale bar: 100 μm)) have a statistical distribution of width σ and mean μ. This distribution can be reconstructed by plotting the single pixel relative error (RE) from 1000 acquired images at the same light intensity (left inset: RE as a function of the number of images or samples; right inset: histogram of the RE values). (**b,c**) Via calibration data of the acquisition sensor, statistically raw-equivalent unprocessed images (with same σ and μ) are generated. (**d**) Image compression is performed on raw data: the differences between the 8-bit, 10:1 JPEG, 7:1 DP compressed formats and the raw image (∆), normalized to the per-pixel noise σ(∆/σ), indicate the artifacts induced by compression on the original image. The displayed image portions correspond to the dotted rectangle in (**a**). (**e**) A SL model, trained on raw data, is used to classify single pixels into two classes A and B. In this work, a Random Forest algorithm classifies pixels according to cell (A) and background (B) classes and produces cell segmentation masks. The model is tested on the statistically raw-equivalent synthetic data to determine the standard deviation ${\upsigma }_{\mathrm{raw}}$ associated to a certain parameter ${\upchi }_{raw}$. The model also predicts a value of the considered parameter from the compressed data ${\upchi }_{\mathrm{c}}$. To verify the statistical impact of image compression, the value of ${\upchi }_{\mathrm{c}}$ is compared to ${\upchi }_{\mathrm{raw}}$ via the standard score $\epsilon =\frac{{\upchi }_{\mathrm{raw}} - {\upchi }_{\mathrm{c}}}{{\upsigma }_{\mathrm{raw}}}$. If $\left|\upepsilon \right|>1,$ SL predictions on the compressed image exceed the statistical variability stemming from raw data noise. The figure has been generated via Python 3.7.3, the Trainable Weka Segmentation plugin (v3.3.1) in ImageJ 2.3.0/1.53f., and Microsoft PowerPoint 16.53.

Raw data, typically obtained through a digitization operation on a physical sample via an acquisition instrument, are intrinsically affected by the noise associated with the acquisition process. When an optical sensor is used, raw data variability is mainly provided by the quantum noise of the photons hitting the sensor, as well as by the electronic noise^21^. Hence, if one performs a sample acquisition under stabilized illumination conditions, as shown in Fig. 1a, the acquired raw images are not identical and the raw pixel values display a statistical distribution of average μ and width σ (the standard deviation associated to the per-pixel noise).

Raw statistics could be in principle determined by repeating and averaging the acquisition of the same image several times. However, these tests are often hard to be carried out in a microscopy laboratory, because of sample variability, non-stationary illumination conditions, mechanical instabilities, as well as computational limitations.

### Sensor calibration and measurement of statistical distortions of SL predictions

The first step of our method consists in calibrating the microscope’s imaging sensor to determine an accurate mapping between light intensities and per-pixel noise. This is performed by sending to the sensor variable intensities from a homogeneous and controlled light source (see Supplementary Material).

Secondly, using the sensor-dependent calibration data (Fig. 1b), statistically raw-equivalent images are numerically generated from a single raw image by replacing the original pixel value with a random one satisfying the raw pixel value statistical distribution (Fig. 1c).

Thirdly, a classification SL model is trained on raw data and provides predictions on a certain parameter $\upchi$. By testing the model on the synthetic images (Fig. 1e), the prediction spread ${\upsigma }_{\mathrm{raw}}$ associated to $\upchi$ can be determined similarly to a Monte-Carlo simulation.

As a final step, we evaluate whether image compression (Fig. 1d) leads to predictions exceeding the original predictive uncertainty ${\upsigma }_{\mathrm{raw}}$. To this end, the difference between the values of a parameter $\upchi$ without (${\upchi }_{\mathrm{raw}})$ and with compression (${\upchi }_{\mathrm{c}}$) is compared to ${\upsigma }_{\mathrm{raw}}$ by using, as a figure of merit, the standard score defined as:

$$\epsilon =\frac{{\upchi }_{\mathrm{raw}} - {\upchi }_{\mathrm{c}}}{{\upsigma }_{\mathrm{raw}}}$$

A standard score $\upepsilon$ such that $\left|\upepsilon \right|<1$ indicates that the statistical variability of SL predictions is smaller than that stemming from the natural noise of raw micrographs, defining therefore a general criterion for tolerability of statistical distortions induced by compression.

### 2D cell segmentation

To demonstrate our method, we perform SL-based cellular segmentation tasks on 2-dimensional (2D) image datasets, obtained through phase-contrast (PC) microscopy, as well as 3-dimensional (3D) datasets obtained via light-sheet (LS) microscopy and optical projection tomography (OPT). By increasing the dimensionality of the raw dataset, we aim at observing how the complexification of raw data noise impacts the SL predictive uncertainty.

**Figure 2:**
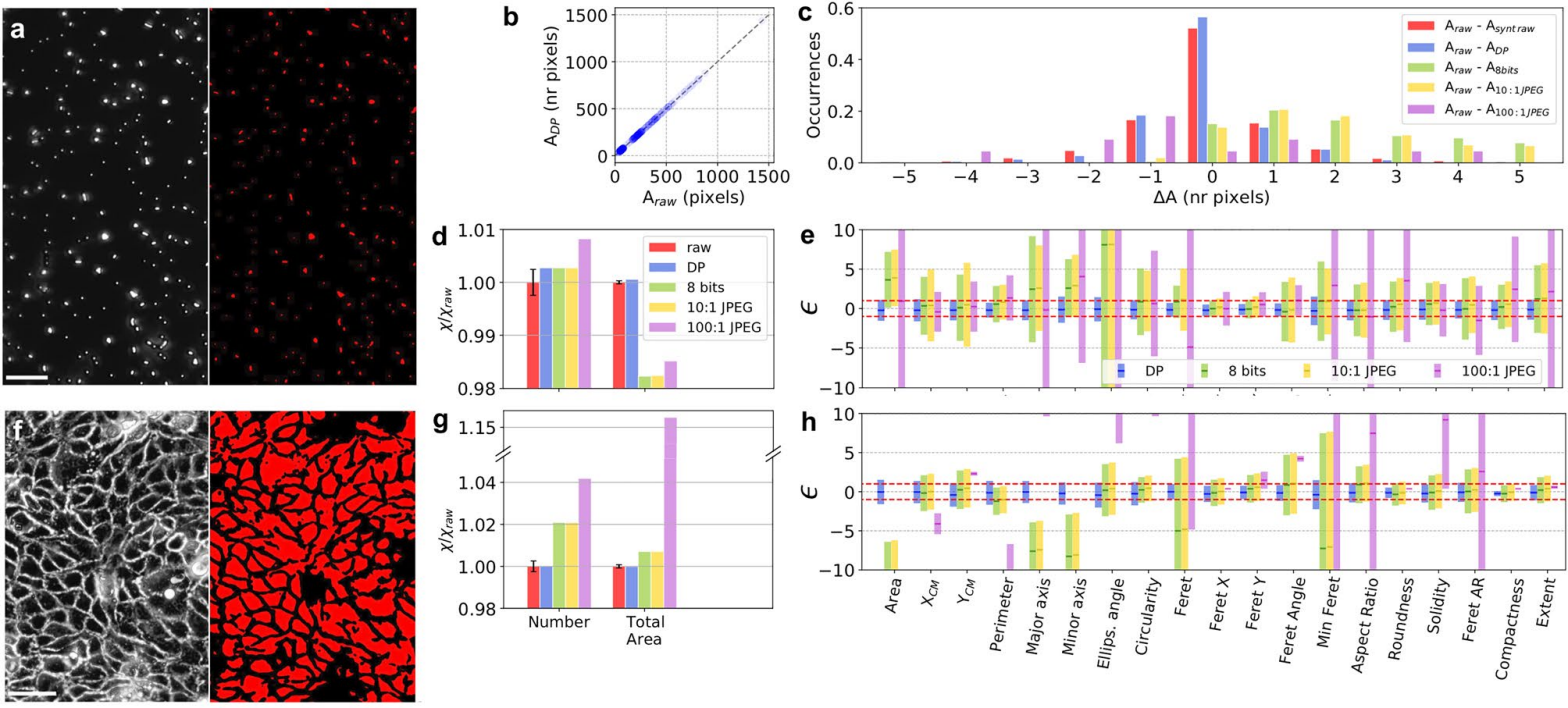
Statistical distortions induced by compression in 2D segmentation tasks in PC microscopy. (**a**) Micrograph of microspheres (scale bar: 20 μm) with corresponding segmentation mask. (**b**) Area of each segmented object obtained from the raw (A_raw_) and the corresponding 6.7:1 DP compressed image (A_DP_) for all objects. **c** Histogram of the difference in the single object area (∆A) determined from the raw and the statistically raw-equivalent images (A_raw_–A_synt raw_), the DP (A_raw_–A_DP_), the 8-bit (A_raw_–A_8bit_), the 10:1 JPEG (A_raw_–A_10:1 JPEG_) and the 100:1 JPEG file (A_raw_–A_100:1 JPEG_). (**d**) Values of the parameters associated to the whole segmented image, such as number of objects and total segmented area, obtained from the DP, the 8-bit and the JPEG segmented images and normalized to the raw value. The error bars on the raw values are calculated from the standard deviation of the values obtained from the synthetic raw images. (**e**) Average of the standard score $\upepsilon$ of 19 parameters associated to the single segmented objects. Red dotted lines correspond to $\varepsilon$ =  ± 1. (**f**) PC Micrograph of MPK cells (scale bar: 50 μm) with segmentation mask, down-sampled to 8-bits and compressed into 6.1:1 DP, 10:1 and 100:1 JPEG formats. (**g**,**h**) Same as (**d**,**e**) for the MPK cells. The figure has been generated via Python 3.7.3, the Trainable Weka Segmentation plugin (v3.3.1) in ImageJ 2.3.0/1.53f, and Microsoft PowerPoint 16.53.

We first consider a cell segmentation task on PC images of microspheres, as well as mouse kidney collecting duct (MPK) cells. A random forest (RF) algorithm^22^, taking decisions on the basis of morphological spatial features, is trained via manual pixel annotations performed on the raw images (details in the Methods section). After producing a segmentation map, we estimate parameters related to the whole image, as well as specific to single segmented objects. After calibration of the acquisition camera, we numerically generate a set of 10 statistically raw-equivalent images and determine the predictive uncertainty ${\upsigma }_{\mathrm{raw}}$ of the considered parameters as the standard deviation of the values obtained with this dataset. The segmented mask obtained from an image of microspheres is shown in Fig. 2a. In Fig. 2d, we compare the total number of objects N_tot_ and the total segmented area A_tot_ predicted from the raw image with those obtained from the corresponding 7:1 DP, 8-bit, 10:1 and 100:1 JPEG file. The difference in N_tot_ with respect to the raw value is in all cases less than 1% ($\epsilon \approx 1$). In contrast, the predicted value of A_tot_ in the 8-bit and JPEG cases ($\epsilon >50$) shows a 2% deviation with respect to the raw result, while only 0.1% variation in the DP case ($\epsilon =-2$).

We then perform a similar analysis on 19 different morphological parameters estimated for each segmented object (area, coordinates of the center of mass (X_*CM*_, Y_*CM*_), perimeter, major axis, minor axis, ellipsoid angle, circularity, Feret, Feret X, Feret Y, Feret angle, Minimum Feret, aspect ratio, roundness, solidity, Feret aspect ratio, compactness, and extent)^23^. For a single object in the raw image, we identify the same object in all synthetic and compressed images by searching for that with the same center coordinates. When this is not possible (around 10% of the cases), we identify the object located at the minimum Euclidean distance with respect to the one considered in the raw image. We reject for the analysis the pairs of objects whose centers are further than 3 pixels (few per cent of the total number of objects).

The inset of Fig. 2b shows a linear trend between the single-object area estimated from the raw (A_raw_) and the DP (A_DP_) mask. The groups of points accumulating along the diagonal correspond to aggregates of microspheres. The mean and the standard deviation of the distribution of the difference in the single-object area (∆A), obtained from the synthetic raw-equivalent images with respect to the raw ones, are in good agreement with the those obtained for the DP format. However, a clear shift of around 3 pixels and a larger spread of the distribution of ∆A is observed in the 8-bit and JPEG cases (Fig. 2c and Table 1).

**Table 1:** Parameters of the distribution of the difference in the single-object area (∆A) obtained from the synthetic and compressed PC microspheres’ image with respect to the raw format (Fig. 2c).

	${\mu }_{\Delta A}$(nr pixels)	${\sigma }_{\Delta A}$(nr pixels)
Synthetic raw-equivalent images	0.1	1.1
DP	− 0.1	1.0
8 bits	3.0	3.4
10:1 JPEG	3.1	3.4
100:1 JPEG	2.9	10.3

In Fig. 2e, we plot the standard scores $\epsilon$ for all parameters averaged over all objects. All scores for the DP format are close to zero and have standard deviations (indicated by error bars) of the order of 1, showing that alterations induced by the DP compression can be considered statistically equivalent to those produced by the intrinsic noise of the raw images. In contrast, the distribution of the standard scores is larger than the [− 1, 1] interval, in some cases of up to 10 standard deviations, with mean values far from 0 for almost all parameters in the 8-bit and JPEG cases. These results imply that predictions on the 8-bit and JPEG files exceed the predictive uncertainty provided by the raw statistical noise. Statistical distortions of predictions on JPEG files are similar to the 8-bit case when the 10:1 factor is used and deviate dramatically (more than 10 standard deviations for almost all parameters) from the raw ones as the compression ratio increases.

The same analysis is performed on a PC micrograph of MPK cells with high confluence and high mean pixel intensity (Fig. 2f). In these experimental conditions, the good agreement between the DP and the raw results is confirmed. We observe a 1–2% deviation in the values of A_tot_ and N_tot_ in the 8-bit and 10:1 JPEG cases (Fig. 2g). As concerns the single object parameters (Fig. 2h), the statistical equivalence of predictions on raw and DP data is confirmed despite the more complex cell spatial configuration. In contrast, predictions on single-object parameters are significantly altered in the 8-bit pixel depth and JPEG cases. This effect turns out to be more relevant for the MPK cells than for the microspheres’ image. Indeed, while in the case of the microspheres almost all segmentation parameters turn out to have a mean value around zero (Fig. 2e), in the case of the MPK cells the mean of almost all parameters strongly deviates from zero (Fig. 2h). We attribute this result to the fact that in the MPK cells’ image the objects to segment are large in terms of number of pixels and very dense. In contrast, the microspheres’ micrograph is characterized by very small objects and large background regions. As segmentation models depend on the loss of information at the boundaries of the objects to segment, statistical distortions due to compression are much more evident in the MPK cells’ case.

### 3D cell segmentation

We then challenge our method on more complex segmentation tasks involving 3D datasets produced by LS microscopy and OPT.

**Figure 3:**
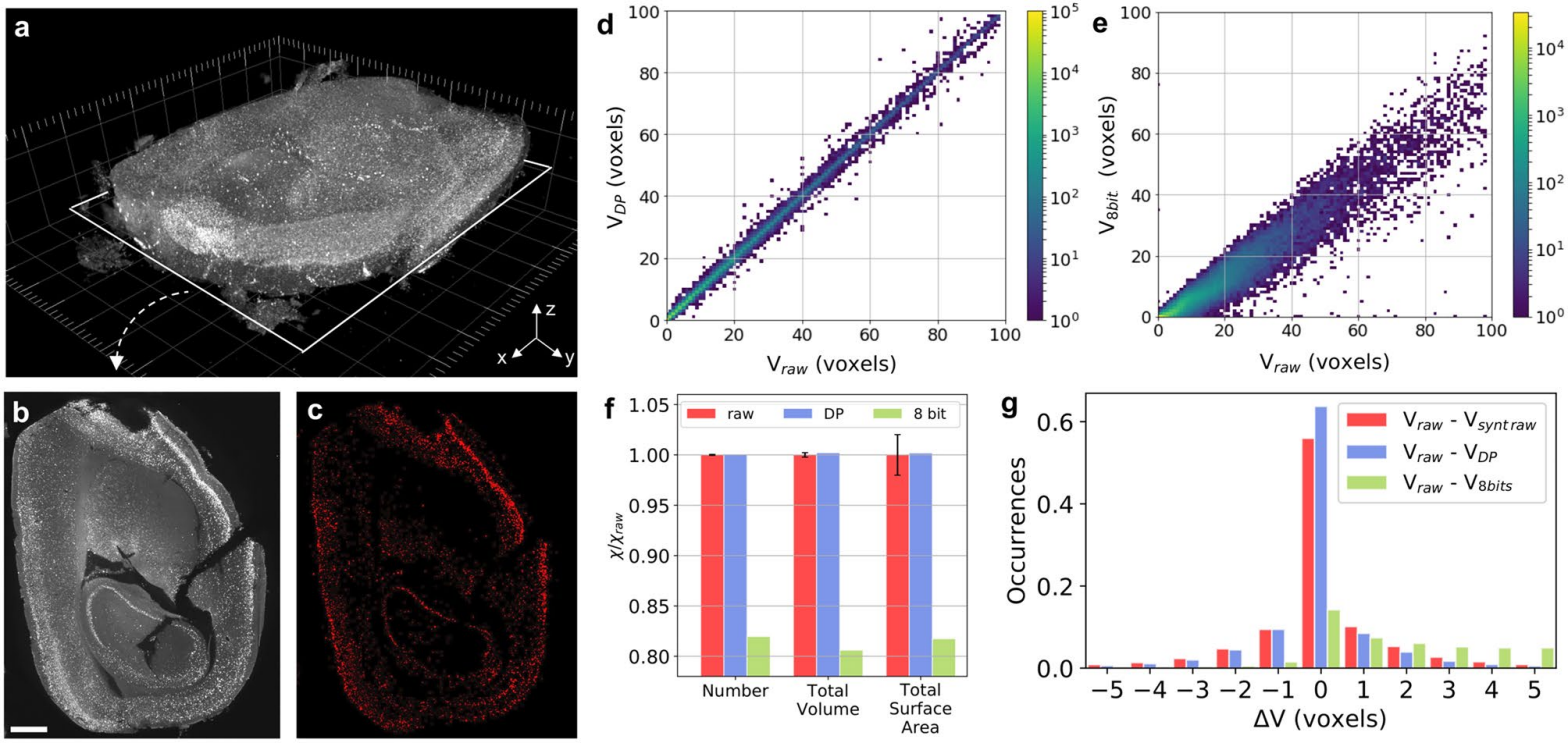
Statistical distortions induced by compression in 3D segmentation tasks in LS microscopy. (**a**) 3D LS image composed of 550 slices from a portion of a mouse brain obtained after *c-fos* staining performed to identify neuronal nuclei (1 voxel = 5.26 μm × 5.26 μm  × 5 μm  = 138 μm^3^). (**b**) Single slice of the 3D image (scale bar: 1 mm) (**c**) Corresponding segmentation mask. (**d,e**) 2D histogram comparing the volume of the same objects in the raw (V_raw_) and the 7.3:1 DP 3D compressed image (V_DP_) (**d**), as well as the 8-bit 3D image (V_8 bits_) (**e**). (**f**) Values of the parameters associated to the whole segmented image, such as the number of objects, total segmented volume and surface area, obtained from the DP and 8-bit segmented image, normalized to the raw value. The error bars on the raw values are calculated from the standard deviation of the values obtained from the synthetic 3D images. (**g**) Histogram of the difference in the single object volume (∆V) calculated from the raw and the statistically raw-equivalent images (V_raw_–V_synt raw_), the DP (V_raw_–V_DP_), and the 8-bit 3D image (V_raw_–V_8bit_). The figure has been generated via iLastik 1.3.2, Python 3.7.3, and Microsoft PowerPoint 16.53.

By using of a RF voxel classification algorithm, we perform segmentation of neuronal nuclei in a portion of a mouse brain after c-*fos* antibody staining, imaged by a mesoscale Selective Plane Illumination LS Microscope (mesoSPIM) (Fig. 3a–c)^24^ and providing a 4.3 GB raw dataset. To obtain the 3D segmented volumes, a threshold criterion is applied to the 3D probability map obtained from the trained ML model. We perform these tests only with the DP method (7.3:1 compression ratio), and 8-bit down-sampling, as JPEG formats are not generally utilized in these fields.

As shown in Fig. 3f, the number of segmented nuclei N_tot_ (around 190′000), as well as the total segmented volume V_tot_ and surface area SA_tot_ calculated from the DP dataset, display a discrepancy of 0.1–0.2% with respect to the values obtained from the raw segmented 3D image ($\epsilon =-1.6, -0.9,-0.1$ for N_tot_, V_tot_ and SA_tot_ respectively) and are perfectly compatible with the spread shown by the synthetic images. In contrast, for all global parameters, the 8-bit conversion provides 15–20% discrepancy, much larger than in the 2D case ($\epsilon =366, 87, 9$ for N_tot_, V_tot_ and SA_tot_ respectively).

We then compare the volume of the single objects segmented from the raw and the corresponding compressed datasets. Also in this case, we find the same objects in the different 3D images by using their center coordinates and their Euclidean distance, as in PC segmentation tests. The 2D histogram in Fig. 3d compares the volume in voxels of each single object identified in the raw (V_raw_) and in the DP dataset (V_DP_). The accumulation of points along the diagonal of the histogram in Fig. 3d reveals the agreement of the predictions obtained with the raw and the DP datasets. The distribution of single-volume differences ∆V provided by the DP stack (Fig. 3g and Table 2) is in good agreement with that obtained from the synthetic raw files. Interestingly, the 8-bit conversion alters dramatically the distribution of ∆V (Fig. 3e). Indeed, the distribution of ∆V in the 8-bit case is not centered around zero and has a much larger spread with respect to the distribution provided by the synthetic raw images (Fig. 3g and Table 2). This type of statistical distortion is similar to that obtained for the distribution of ∆A after segmentation of the PC microspheres’ image (Fig. 2c,e). Given the large discrepancy observed for the global parameters, the 8-bit conversion seems affecting the predictions of the segmentation models in a more complex way with respect to the 2D case.

**Table 2:** Parameters of the difference in the single-object volume (∆V) obtained from the synthetic and compressed LS 3D image with respect to the raw format (Fig. 3g).

	${\mu }_{\mathrm{\Delta V}}$(nr voxels)	${\sigma }_{\mathrm{\Delta V}}$(nr voxels)
Synthetic raw-equivalent images	0.1	2.7
DP	− 0.1	2.2
8 bits	7.3	6.4

**Figure 4:**
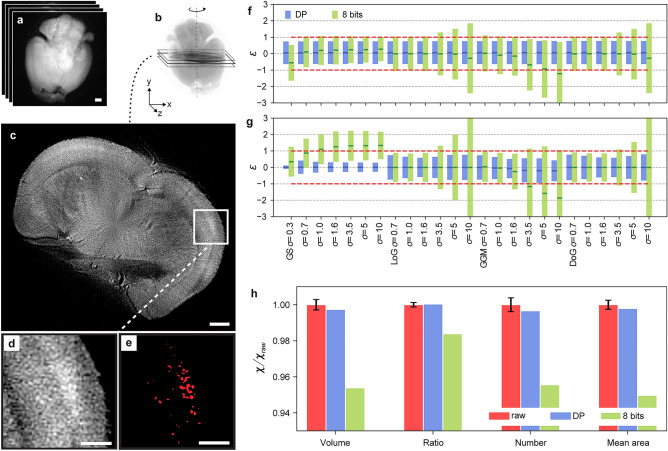
Statistical distortions induced by compression in 3D segmentation of amyloid Alzheimer plaques imaged via OPT. (**a**–**c**) Typical OPT pipeline. Fluorescent projections of an organ at different angles are used to reconstruct its 3D image via an inverse Radon transform. Transverse slices are used for physiological analysis (scale bar: 1 mm). (**d**) Close-up of a reconstructed slice showing amyloid plaque deposition in the mice brain. (**e**) Segmented mask of amyloid plaques (scale bar: 300 μm). (**f,g**) Single pixel standard scores associated to the four spatial operators used for training (Gaussian Smoothing (GS), Laplacian of Gaussian (LoG), Gaussian Gradient Magnitude (GGM) and Difference of Gaussians (DoG)) applied with different sizes of the gaussian kernel σ. Standard scores of these operators are calculated on the projections (**f**) and the reconstructed image (**g**) obtained from the 7.8:1 DP compressed and the 8-bit converted 3D image. Red dotted lines correspond to $\varepsilon$ =  ± 1. (**h**) Normalized values of amyloid plaque characteristics associated to the whole dataset, obtained from the raw (red), the DP compressed (blue) and the 8-bit converted datasets (green). The error bars on the raw values are determined via the synthetic statistically raw-equivalent 3D images. The figure has been generated via iLastik 1.3.2, Python 3.7.3 and Adobe Illustrator 25.0 (2021).

We then adopt our compression tolerability method on a pre-clinical application, based on the estimation of amyloidosis in a middle-aged mouse brain affected by Alzheimer’s disease via Optical Projection Tomography (OPT) imaging. OPT is well suited to image mesoscopic centimeter-sized biological specimens, such as organs, and represents the optical equivalent of computed tomography: fluorescent projection images are captured at different angles around the specimen and the 3D image of the organ is reconstructed via a Filtered Back Projection (FBP) algorithm using an inverse Radon transform (Fig. 4a–c)^25^. Compared to the segmentation performed on LS microscopy data, an image reconstruction step is introduced before the AI testing, making more complex the propagation of the original noise of the raw projections through the AI pipeline. To obtain the amyloid plaques segmentation mask on the raw and compressed 3D images, we adopt the same type of RF algorithm used for the LS microscopy data to classify every voxel of the reconstructed images and apply a threshold criterion on the 3D probability map (namely 0.7 for brain anatomy and 0.5 for amyloid plaques) according to a previous study^26^. A close up of a slice of a reconstructed diseased mouse brain and the corresponding plaques segmentation mask are shown in Fig. 4d,e, respectively. In this experiment, to quantify the SL predictive uncertainty, we simulate a dataset of 10 statistically raw-equivalent projections of the mouse brain. In this case, we first apply our method to the spatial operators used for model training by considering the 7.8:1 DP compressed and the 8-bit converted projections datasets, and by calculating standard scores of these operators averaged over all projection pixels. As shown by Fig. 4f, the averaged standard scores belong for all parameters to the [-1,1] interval in the DP case. In contrast, the 8-bit conversion shows in general larger discrepancies for an increasing gaussian smoothing. Similar results are found when the considered processing operations are performed after the 3D reconstruction (Fig. 4g). In this case, the averaged standard scores of the gaussian smoothing operators applied over all voxels of the reconstructed 3D images are around 0 because smoothing is already performed in the applied FBP algorithm^26^.

Finally, we compare the sensitivity of the quantitative analysis of amyloidosis in the mouse brain to DP compression and 8-bit conversion. Given the complete 3D prediction map, we compute the values of the standard scores for four plaque parameters: the total volume of plaque, the plaque load (the ratio of plaque volume to the total organ volume), the total plaque count and the plaque mean volume. The results, shown in Fig. 4h, indicate that global segmentation parameters are conserved upon DP compression. In contrast, the 8-bit conversion provides around 5% deviation with respect to the raw values, confirming the alteration of the raw predictions also in this case.

## Discussion

In this work, we show the statistical nature of the distortions induced by image compression on the outcomes of SL tasks in optical microscopy. We present an experimental method capable of quantifying these statistical distortions, relying on determining the SL predictive uncertainty from the intrinsic statistical noise of raw data. As raw noise is unavoidable, our approach sets a lower bound to the predictive uncertainty in SL-assisted decision-making processes.

We show that 16-to-8 bits pixel depth reduction and JPEG compression can alter SL outcomes by more than 10 standard deviations. Interestingly, these distortions are more relevant in 3D applications: the use of 8-bit conversion brings to 5% and 15% prediction change in OPT and LS datasets segmentation, respectively, exceeding raw predictions by many standard deviations. The different distortions in the two 3D cases are probably related to the different propagation of the raw data noise through the processing pipeline. In contrast, we observe that alterations induced by the DP compression can be considered as statistically equivalent to those provided by the raw noise in both 2D and 3D cases. By respecting the raw pixel value statistics and providing compressed images with size reduced by a factor up to 10, the DP image format represents a valuable solution to computational and data management challenges associated to computer-vision automated tasks in microscopy.

In this work, we also measure the statistical distortions of the predictions of a pre-clinical cell segmentation task in OPT. Our results indicate that the reliability of scientific outcomes obtained in diagnostic applications can be compromised by image compression in a non-negligible way.

Finally, our work highlights the importance to preserve raw pixels statistics in image processing pipelines prior to the application of a SL model. Respecting raw statistics allows to achieve the minimum prediction spread of the model, corresponding to that obtainable without processing.

When used to quantify predictions’ distortions induced by lossy compression techniques, that do not preserve raw statistics, our method can still provide a criterion of tolerability of the adopted compression. However, in this case, as the raw predictive uncertainty depends on the SL algorithm and its optimization features, our technique cannot prevent dependencies between the AI model and the lossy compression.

We expect our method to be generalizable to any field where acquisition devices can be calibrated and raw data are processed before AI use.

## Methods

### Phase contrast (PC) microscopy

For PC microscopy measurements, we used two inverted PC microscopes (Axiovert 40C and Axiovert 25, Carl Zeiss Jena GmbH) equipped with 5×, 10× and 20× objectives and a calibrated CMOS camera (CM3-U3-31S4M-CS, Sony). Samples of carboxylate microspheres of 500 nm diameter (Polysciences) were obtained by deposing 5 μL of an aqueous solution with a concentration of 3.6·10^8^ particles/mL on a 170 μm thick glass slide. After solvent evaporation, PMMA at 0.1 g/mL was added to the sample, then centrifugated and dried. Microspheres were imaged with a 20 × objective and used for segmentation tests, as well as for the measurement of the microscope’s point spread function (PSF). Two cell lines with variable confluence have been cultured: human neural stem cells (HIP) (A3890101, ThermoFisher)^27^ and mpkCCDC14 mouse kidney collecting duct cells (MPK)^28,29^. Both cell lines were cultured and grown on a cell treated plastic surface at a temperature of 37 °C with an air atmosphere enriched with 5% CO_2_. Micrographs of HIP and MPK cells were obtained with a 10 × and 20 × objective, respectively. For the measurement of the modulation transfer function (MTF), we imaged the 1951 USAF test target (R3L3S1P, Thorlabs Inc.) in bright field configuration with the 5 × objective. Segmentation tests have been performed on a microspheres’ and a MPK cells’ image, that have been compressed into different formats (file sizes for all tested formats are shown in Table 3).

**Table 3:** Size of the datasets used for the segmentation tests performed in this work.

	Microscopy technique	RAW (16 bits)	8 bits	DP	10:1 JPEG	100:1 JPEG
Microspheres (Fig. 2a)	PC	2.4 MB	1.2 MB (2:1)	0.36 MB (6.7:1)	0.25 MB (10:1)	21 kB (114:1)
MPK cells (Fig. 2f)	PC	6.3 MB	3.1 MB (2:1)	1.0 MB (6.1:1)	0.63 MB (10.1)	64 kB (98:1)
Mouse brain after c-fos staining (Fig. 3a,b)	LS	4.3 GB	2.1 GB (2:1)	0.6 GB (7.3:1)		
Mouse brain with amyloid plaques (Fig. 4c)	OPT	0.5 GB	0.25 GB (2:1)	64 MB (7.8:1)		

To perform these tests, we have trained machine learning models via the trainable Weka segmentation ImageJ plug-in^30^, combining a collection of machine learning algorithms with a selection of image features to produce pixel classification. As a classifier, we used the FastRandomForest, a multi-threaded reimplementation of Random Forest created by Fran Supek, which optimizes speed and memory usage^30^. This classifier is initialized with 200 trees and 2 random features per node. Training of this classifier is performed by manually annotating pixels in the original raw image according to two classes of identical weight: cell and background. The selected training features are: gaussian blur, hessian, sobel filter and difference of gaussians (details on the training procedure are provided in the Supplementary Information). To analyze the segmented masks, we used the “Extended Particle Analyzer” macro of the Biovoxxel toolbox in ImageJ^23^, allowing to analyze the segmented objects according to a large variety of morphological parameters, shape descriptors and angle orientations. As the RF model was trained on a single 16-bit raw image, the synthetic images and the DP compressed were automatically suited to be tested by the model, while the 8-bit and JPEG files needed to be upsampled to 16-bit depth.

### Light-sheet (LS) microscopy

For LS microscopy measurements, we used a home-built mesoscale single-plane illumination microscope^24^. This setup consists of a dual-sided excitation path using a fiber-coupled multiline laser combiner (405, 488, 561 and 647 nm, Toptica MLE) and a detection path comprising a 42 Olympus MVX-10 zoom microscope with a 1 × objective (Olympus MVPLAPO 1x), a filter wheel (Ludl 96A350), and a calibrated scientific CMOS camera (Hamamatsu Orca Flash 4.0 V3). The excitation paths also contain galvo scanners for light-sheet generation and reduction of shadow artifacts due to absorption of the light-sheet. In addition, the beam waist is scanned using electrically tunable lenses (ETL, Optotune EL-16-40-5D-TC-L) synchronized with the rolling shutter of the camera. Sample was illuminated by one of the two acquisition paths. Image acquisition was done using custom software written in Python 3. Z-stacks were acquired at 5 μm spacing with a zoom set at 1.25X resulting in an in-plane pixel size of 5.26 μm (2048 × 2048 pixels). Excitation wavelength of the c-*fos* antibody was set at 647 nm with an emission filter LP 663 nm bandpass filter (BrightLine HC, AHF).

Concerning the sample preparation, mice were perfused with 4% PFA and tissue was post-fixed overnight in 4% PFA. Mouse brain were prepared for imaging following the iDISCO procedure described by Renier et al.^31^. To visualize a reporter of neuronal activity (c-*fos*), a c-*fos* antibody (synaptic System Anti c-*Fos* CN226003) was used to label the brain 1:2000 (0.25 ug/ml). This was coupled to an anti-rabbit Alexa Fluor-647 (far-red spectrum) (5ug/ml). After clearing, brains were immersed in a 10 × 20 × 45 mm quartz cuvette filled with DiBenzyl Ether (RI 1.56).

For PSF measurements, fluorescent Tetraspeck microspheres 0.1 μm were diluted into 1% agarose. Excitation wavelength was set at 488 nm with an emission 530/40 nm bandpass filter (BrightLine HC, AHF).

3D Segmentation tests have been performed on the dataset obtained by imaging the mouse brain after *c-fos* antibody treatment (sizes of the dataset in the raw and tested compressed formats are shown in Table 3). To perform these tests and segment the c-*fos* positive neuron nuclei, we have trained a voxel classification model via the open-source image analysis software iLastik^32^. As a classifier, we used a Random Forest algorithm initialized with 100 trees that are built by randomly picking features among four spatial operators (Gaussian Smoothing (GS), Laplacian of Gaussian (LoG), Gaussian Gradient Magnitude (GGM) and Difference of Gaussians (DoG)) applied with a varying gaussian kernel σ.

Training of this classifier is performed by manually annotating some voxels of the 3D raw image according to three classes of identical weight (anatomy, c-*fos* positive nuclei, and background).

The trained algorithm predicted the class of the remaining voxels in the full image. To do so, the software attributed to each voxel a vector of computed features and provided a 3D probability map where the value of each voxel corresponded to the likelihood to be a c-*fos* positive neuronal nucleus. The final nuclei segmentation was then realized by applying a threshold value of 0.5 to the 3D probability map. As done for the 2D segmentation tests, the RF model was trained on a single 16-bit raw image stack, while the 8-bit stacks are upsampled to 16-bit depth.

### Optical projection tomography (OPT)

For OPT measurements, we processed datasets of full intact mouse brains previously studied in^26^ using an SL approach to quantify amyloidosis of an Alzheimer’s disease mouse model. The epi-fluorescent image projections were acquired with a custom mesoscopic OPT setup consisting of a calibrated CMOS camera (ORCA-Flash 4.0 V2, Hamamatsu) coupled to a 300-mm achromat objective lens providing 0.5X magnification of the sample^33^. The sample was mounted on a motorized rotation stage allowing for projection acquisitions over 360 degrees by steps of 0.3 or 0.9 degrees in approximately five minutes. The sample fluorescent signal was excited by a 420-nm LED light source illuminating the whole organ. In this configuration, the OPT setup had an isotropic pixel-limited resolution of approximately 50 μm over the whole organ, due to the physical pixel size of the camera. Each set of projections (1024 × 1024 × 1200 matrix for 1200 projections) were previously saved as uncompressed 16-bit stacked .tif files. The 3D reconstruction of the sample was achieved by applying a filtered back-projection (FBP)^34^ to the raw and compressed projection sets with the Matlab iRadon function. To do so, we follow the same procedure as previously described in^33^. After reconstruction, the 3D image volumes were cropped along the three dimensions in order to remove background contribution and conserve the brain signal only. The intensity of each voxels was normalized over the volume using Fiji^35^ and its contrast enhancement tool before SL segmentation to accommodate for differences in dynamic range.

The plaque segmentation tests relied on the same classification workflow realized with iLastik^32^ for the segmentation tests on LS datasets. In the case of the OPT dataset (sizes of the dataset in the raw and tested compressed formats are shown in Table 3), the three classes manually labeled in the training step were plaque, anatomy, and background. 12 slices (2D) from the full mouse brain were manually annotated (brain tissues and amyloid plaques), a part of them were used to train the RF algorithm, while the rest served as a test set. The final plaque segmentation and quantification was then realized by applying a threshold value of 0.5 to the 3D plaque probability map. This pipeline was the same as that provided in a previous study^26^.

## Supplementary Information

Supplementary Information.

## References

[1] Sommer C, Gerlich DW (2013). Machine learning in cell biology-teaching computers to recognize phenotypes. J. Cell Sci..

[2] Van Valen DA (2016). Deep learning automates the quantitative analysis of individual cells in live-cell imaging experiments. PLoS Comput. Biol..

[3] von Chamier L, Laine RF, Henriques R (2019). Artificial intelligence for microscopy: What you should know. Biochem. Soc. Trans..

[4] Vu QD (2019). Methods for segmentation and classification of digital microscopy tissue images. Front. Bioeng. Biotechnol..

[5] Bychkov D (2018). Deep learning based tissue analysis predicts outcome in colorectal cancer. Sci. Rep..

[6] Xu Y (2017). Large scale tissue histopathology image classification, segmentation, and visualization via deep convolutional activation features. BMC Bioinform..

[7] Laanait N, Zhang Z, Schlepütz CM (2016). Imaging nanoscale lattice variations by machine learning of X-ray diffraction microscopy data. Nanotechnology.

[8] Bulgarevich DS, Tsukamoto S, Kasuya T, Demura M, Watanabe M (2018). Pattern recognition with machine learning on optical microscopy images of typical metallurgical microstructures. Sci. Rep..

[9] Maksov A (2019). Deep learning analysis of defect and phase evolution during electron beam-induced transformations in WS2. NPJ Comput. Mater..

[10] Esteva A (2017). Dermatologist-level classification of skin cancer with deep neural networks. Nature.

[11] Munir K, Elahi H, Ayub A, Frezza F, Rizzi A (2019). Cancer diagnosis using deep learning: A bibliographic review. Cancers.

[12] Zelger P (2018). Three-dimensional localization microscopy using deep learning. Opt. Express.

[13] Möckl L, Roy AR, Moerner WE (2020). Deep learning in single-molecule microscopy: Fundamentals, caveats, and recent developments [Invited]. Biomed. Opt. Express.

[14] Chen H, Engkvist O, Wang Y, Olivecrona M, Blaschke T (2018). The rise of deep learning in drug discovery. Drug Discov. Today.

[15] Stokes JM (2020). A deep learning approach to antibiotic discovery. Cell.

[16] Rivenson Y (2017). Deep learning microscopy. Optica.

[17] Wang H (2019). Deep learning enables cross-modality super-resolution in fluorescence microscopy. Nat. Methods.

[18] Wu Y (2019). Three-dimensional virtual refocusing of fluorescence microscopy images using deep learning. Nat. Methods.

[19] Begoli E, Bhattacharya T, Kusnezov D (2019). The need for uncertainty quantification in machine-assisted medical decision making. Nat. Mach. Intell..

[20] Sanguinetti, B. & Clausen, C. Method and device for steganographic processing and compression of image data. US Patent App. 17/090,577 (2020).

[21] Pence WD, Seaman R, White RL (2009). Lossless astronomical image compression and the effects of noise. Publ. Astron. Soc. Pacific.

[22] Breiman L (2001). Random forrests. Mach. Learn..

[23] Brocher, E.-C. The BioVoxxel Image Processing and Analysis Toolbox. in *Eur. BioImage Anal. Symp., Paris* 336–338 (2013).

[24] Voigt FF (2019). The mesoSPIM initiative: Open-source light-sheet microscopes for imaging cleared tissue. Nat. Methods.

[25] Sharpe J (2002). Optical projection tomography as a tool for 3D microscopy and gene expression studies. Science.

[26] Nguyen D (2019). Supervised learning to quantify amyloidosis in whole brains of an Alzheimer’s disease mouse model acquired with optical projection tomography. Biomed. Opt. Express.

[27] Govindan S, Batti L, Osterop SF, Stoppini L, Roux A (2021). Mass generation, neuron labeling, and 3D imaging of minibrains. Front. Bioeng. Biotechnol..

[28] Bens M (1999). Corticosteroid-dependent sodium transport in a novel immortalized mouse collecting duct principal cell line. J. Am. Soc. Nephrol..

[29] Hasler U, Vinciguerra M, Vandewalle A, Martin PY, Féraille E (2005). Dual effects of hypertonicity on aquaporin-2 expression in cultured renal collecting duct principal cells. J. Am. Soc. Nephrol..

[30] Arganda-Carreras I (2017). Trainable weka segmentation: A machine learning tool for microscopy pixel classification. Bioinformatics.

[31] Renier N (2014). IDISCO: A simple, rapid method to immunolabel large tissue samples for volume imaging. Cell.

[32] Berg S (2019). ilastik: Interactive machine learning for (bio)image analysis. Nat. Methods.

[33] Nguyen D (2017). Optical projection tomography for rapid whole mouse brain imaging. Biomed. Opt. Express.

[34] Kak AC, Slaney M, Wang G (2002). Principles of computerized tomographic imaging. Med. Phys..

[35] Schindelin J (2012). Fiji: An open-source platform for biological-image analysis. Nat. Methods.

