# Supplementary material for: Statistical distortion of supervised learning predictions in optical microscopy induced by image compression
Source: Sci Rep. 2022 Mar 2;12:3464. doi: 10.1038/s41598-022-07445-4 (PMC8891276; doi:10.1038/s41598-022-07445-4)
Supplement: Supplementary file 1 — Supplementary Information. [file 41598_2022_7445_MOESM1_ESM.pdf]

SUPPLEMENTARY INFORMATION

Statistical distortion of supervised learning predictions in optical microscopy induced by image compression

Enrico Pomarico[1*], Cédric Schmidt[1], Florian Chays[1], David Nguyen[2], Arielle Planchette[2], Audrey Tissot[3], Adrien Roux[1], Stéphane Pagès[3,4], Laura Batti[3], Christoph Clausen[5], Theo Lasser[6], Aleksandra Radenovic[2], Bruno Sanguinetti[5], and Jérôme Extermann[1]

[1] HEPIA, HES-SO, University of Applied Sciences and Arts Western Switzerland, Rue de la Prairie 4, 1202 Geneva, Switzerland

[2] Laboratoire de Biologie à l'Echelle Nanométrique, School of Engineering, École Polytechnique Fédérale de Lausanne, CH-1015 Lausanne, Switzerland

[3] Wyss Center for Bio- and Neuroengineering, Geneva, Switzerland

[4] Department of Basic Neurosciences, Geneva Neuroscience Center, Faculty of Medicine, University of Geneva, Geneva, Switzerland

[5] Dotphoton SA, Zeughausgasse 17, 6300 Zug, Switzerland

[6] Max-Planck Institute for Polymer Research, Ackermannweg 10, 55128 Mainz, Germany

[*]email: enrico.pomarico@hesge.ch

Optical calibration of a microscope camera

The pixel value recorded in a microscope image depends both on the signal (mean number of photons impinging on the pixel) and noise. In general, the information content deriving from the signal cannot be distinguished from the entropy due to noise. During camera calibration, we project a series of specific mean photons numbers on the bare sensor pixels, i.e. we inject a signal known in advance, so that the noise model of the camera may be accurately determined. Our procedure is adapted from that present in the EMVA1288 standard[1].

The inside of polytetrafluoroethylene (PTFE) integrating sphere is illuminated by a white LED stable to better than $1/10^5$ over a range of output powers from 0.1 mW to 200 mW (Figure S1). The light intensity is measured through a 1cm x 1cm NIST-traceable photodiode connected to a calibrated 7-digit voltmeter placed on the surface of the sphere. The sensor is placed on the axis of the main 5 cm sphere aperture, at a 1 m distance.

In order to calibrate the camera sensor, 1000 images are acquired for each of 200 different illuminations, that are spaced according to a square-law from complete darkness to sensor saturation. For each pixel we can therefore map each input light level (mean photon number $\langle n \rangle$) to a histogram of the recorded digital pixel values d, as shown in Figure S2a. From this plot, a mathematical model of the sensor response can be formulated, as described in the EMVA1288[1]. In particular, the relation between the standard deviation of the per-pixel noise σ and the pixel value d can be determined (Figure S2b).

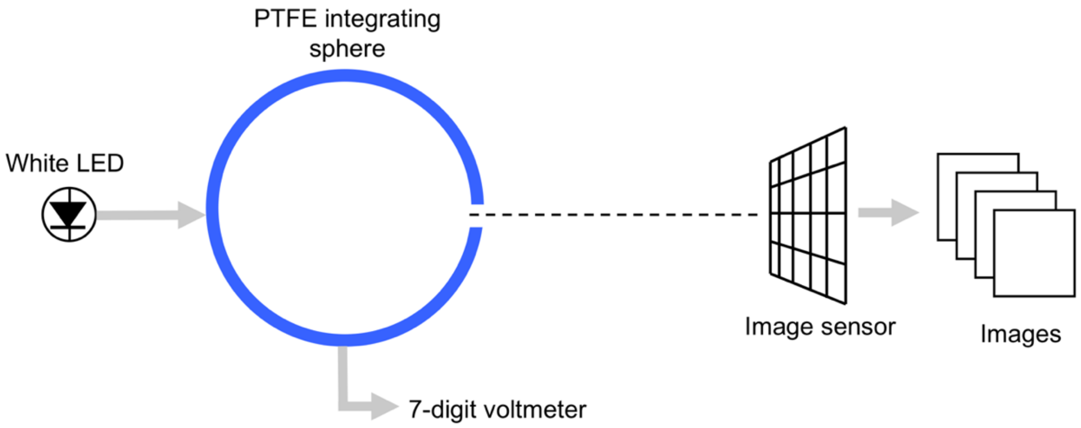

Figure S1 **Experimental setup for microscope cameras calibration**

A PTFE integrating sphere is illuminated by a white LED. A photodiode is connected to a calibrated 7-digit voltmeter. The microscope camera is placed on the axis of the sphere at 1 m from the 5 cm aperture of the PTFE sphere. The figure has been generated via Microsoft PowerPoint 16.53.

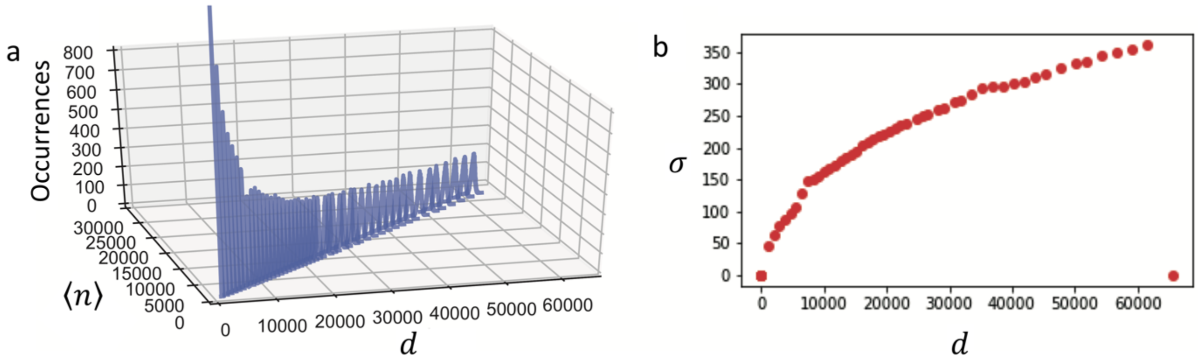

Figure S2 **Calibration curve of a single camera pixel**

a 3D representation of the statistical distribution of a pixel value d for multiple input photon numbers $\langle n \rangle$. For a given $\langle n \rangle$, the number of occurrences is shown together with the digital pixel value d returned by the sensor. **b** Plot of the standard deviation of the per-pixel noise σ as a function of d extracted from the statistical distribution shown in **a**. The figure has been generated via Python 3.7.3 and Microsoft PowerPoint 16.53.

Training of the Random Forest (RF) algorithm for segmentation tests on PC microscopy images

To implement the method described in the present work, different machine learning models have been trained on raw data for different cellular segmentation applications.

As described in the Methods section, in order to segment PC images, we have trained the FastRandomForest algorithm via the Weka segmentation ImageJ plug-in. In this case, classifier training has consisted in manually annotating single pixels of the raw image according to two classes: cell and background.

In the case of the microspheres' micrograph shown in Figure 2a, 592 pixels (0.05% of the total number of pixels) have been annotated for the cell class and 71864 (6%) for the background one. The classifier is initialized with 200 trees and is trained by using 2 random features per node selected between gaussian blur, hessian, sobel filter and difference of gaussians operators. Training provides an out-of-bag error of 0.05%.

To assess the performance of the trained model, we have calculated on a test set the receiver operating characteristic (ROC) and the precision – recall curves (Figure S3), illustrating the diagnostic ability of the binary classifier for a variable discrimination threshold applied to the probability map provided by the model. The test set is based on annotated pixels that have not been used for training: 304 pixels for the cell class and 4301 for the background one.

The ROC curve is created by plotting the true positive rate (TPR) against the false positive rate (FPR) at various threshold settings (Figure S3 left). TPR and FPR are calculated as

$$TPR = \frac{TP}{TP+FN} \quad \text{and} \quad FPR = \frac{FP}{FP+TN},$$

where T(F) stands for true (false) and P (N) for positive (negative). Instead, the precision – recall curve is obtained by plotting the Precision (Pr) parameter with respect to the recall (R) one, defined as

$$Pr = \frac{TP}{TP+FP} \quad \text{and} \quad R = \frac{TP}{TP+FN} = TPR$$

ROC and precision-recall curves for the microspheres' micrograph are shown in Figure S3.

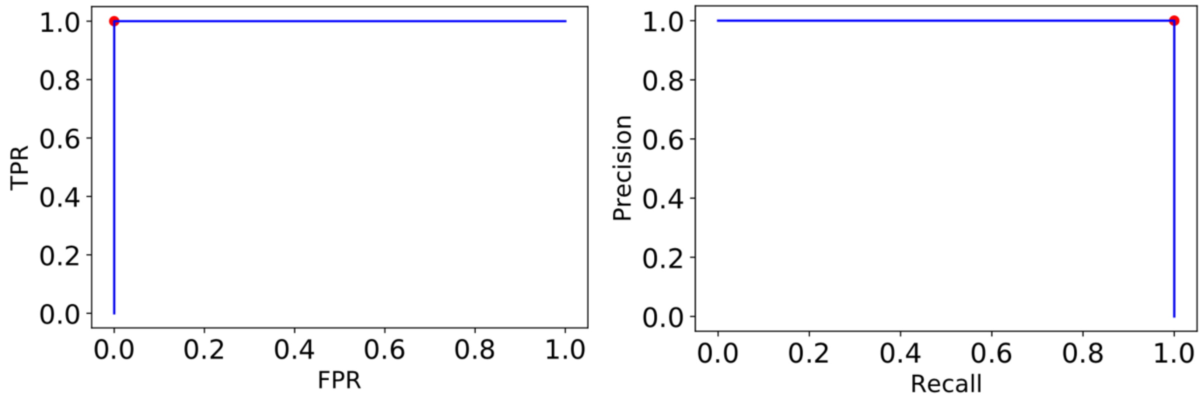

Figure S3 **ROC and precision-recall curve for the cell vs background classification on microspheres PC image.** The 0.5 threshold value is shown in red. The figure has been generated via Python 3.7.3 and Microsoft PowerPoint 16.53.

The confusion matrix at a threshold of 0.5 is provided by the Table S1

	Predicted: Negative	Predicted: Positive
Actual: Negative	4301 (TN)	0 (FP)
Actual: Positive	0 (FN)	304 (TP)

Table S1 Confusion matrix corresponding to a threshold of 0.5 associated to the segmentation of the microspheres' PC micrograph

Corresponding to this confusion matrix, we obtain a value for the Jaccard index (JI), calculated as

$$JI = \frac{TP}{TP+FP+FN},$$

of 100%.

ROC and precision-recall curves, as well as the value of the Jaccard index, confirm the very good performance of the classifier used to segment the microspheres' image.

In the case of the MPK cells' micrograph shown in Figure 2f, training has been performed by using 34130 pixels (1% of the total number of pixels) annotated for the cell class and 8682 (0.3%) for the background one. Training provides an out-of-bag error of 0.13%. The test set consisted of 8277 pixels for the cell class and 5388 pixels for the background. ROC and precision-recall curves calculated on the test set are shown in Figure S4.

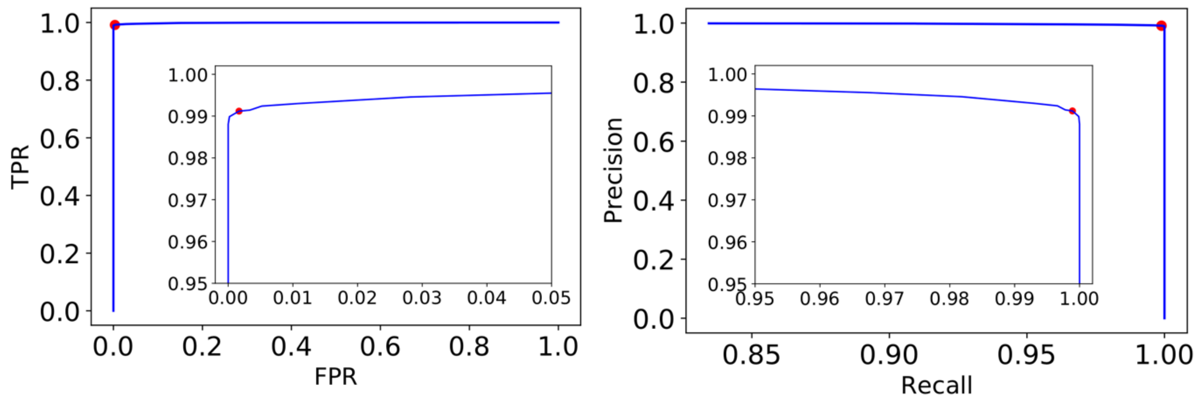

Figure S4 **ROC and precision-recall curve for the cell vs background classification of the MPK cells' PC image.** The 0.5 threshold value is shown in red. The figure has been generated via Python 3.7.3 and Microsoft PowerPoint 16.53.

The confusion matrix at a threshold of 0.5 is given by Table S2

	Predicted: Negative	Predicted: Positive
Actual: Negative	5379 (TN)	9 (FP)
Actual: Positive	73 (FN)	8204 (TP)

Table S2 Confusion matrix corresponding to a threshold of 0.5 associated to the segmentation of the MPK cells' micrograph

and provides a JI of 99%, confirming the good quality of the trained classifier also in the case of the MPK cells' micrograph.

Training of the RF algorithm for segmentation tests with LS microscopy and OPT images

In the case of the voxel classification performed on the LS microscopy dataset (Figure 3a and b), the training set consisted of 586601 voxels (0.05% of the total number of pixels) annotated for the background class, 60599 (0.005%) for the anatomy class, and 230 (0.00002%) for the nuclei one. RF classifier provides an out-of-bag error of 0.034%. ROC and precision-recall curves for the anatomy vs background (Figure S5) and for the nuclei vs anatomy (Figure S6) classifications have been calculated on a separated test set, consisting of 622856 voxels annotated for the background class, 57668 for the anatomy class, and 640 for the nuclei one.

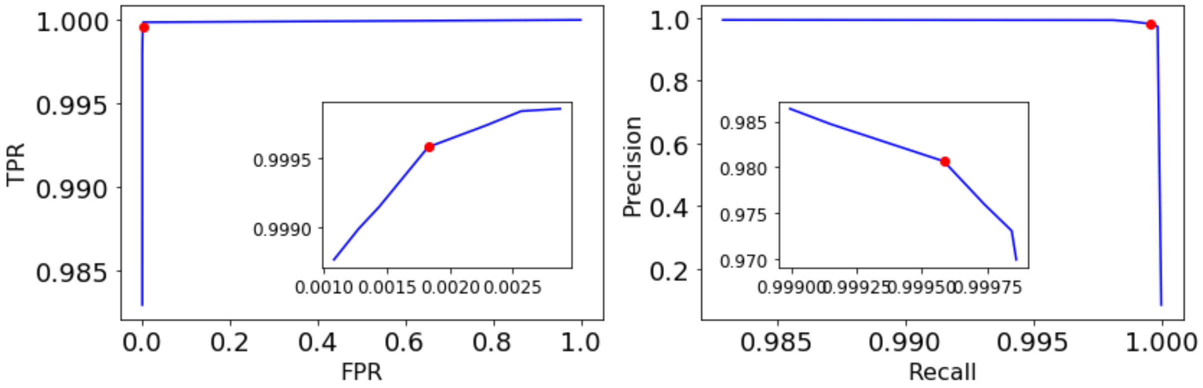

Figure S5 **ROC and precision-recall curve for the anatomy vs background voxel classification of the LS microscopy dataset.** The 0.5 threshold value is shown in red. The figure has been generated via Python 3.7.3 and Microsoft PowerPoint 16.53.

The confusion matrix associated to anatomy vs background classification at a threshold of 0.5, corresponding to a JI of 98%, is given in Table S3

	Predicted: Negative	Predicted: Positive
Actual: Negative	621718 (TN)	1138 (FP)
Actual: Positive	24 (FN)	57644 (TP)

Table S3 Confusion matrix corresponding to a threshold of 0.5 associated to the anatomy vs background classification in the LS microscopy dataset

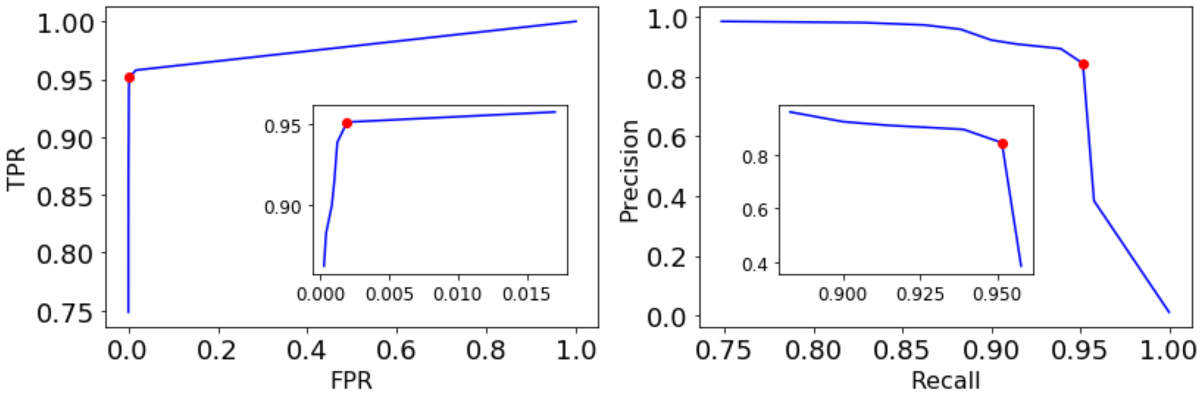

Figure S6 **ROC and precision-recall curve for the nuclei vs anatomy voxel classification of the LS microscopy dataset.** The 0.5 threshold value is shown in red. The figure has been generated via Python 3.7.3 and Microsoft PowerPoint 16.53.

The confusion matrix for the nuclei vs anatomy classification at a threshold of 0.5, which provides a JI of value of 81%, is given by Table S4

	Predicted: Negative	Predicted: Positive
Actual: Negative	57556 (TN)	112 (FP)
Actual: Positive	31 (FN)	609 (TP)

Table S4 **Confusion matrix corresponding to a threshold of 0.5 associated to the nuclei vs anatomy classification in the LS microscopy dataset**

For the classification of amyloid Alzheimer plaques performed on the OPT dataset (Figure 4), the training set consisted of 195290 voxels (0.2% of the total number of pixels) annotated for the background class, 69516 (0.08%) for the anatomy class, and 450 (0.0005%) for the plaques one. RF classifier provides an out-of-bag error of 0.018%. ROC and precision-recall curves for the anatomy vs background (Figure S7) and for the anatomy vs plaques (Figure S8) classifications have been calculated. The test set consisted of 18844 voxels (0.02% of the total number of pixels) annotated for the background class, 10723 (0.01%) for the anatomy class, and 250 (0.0003%) for the plaques one.

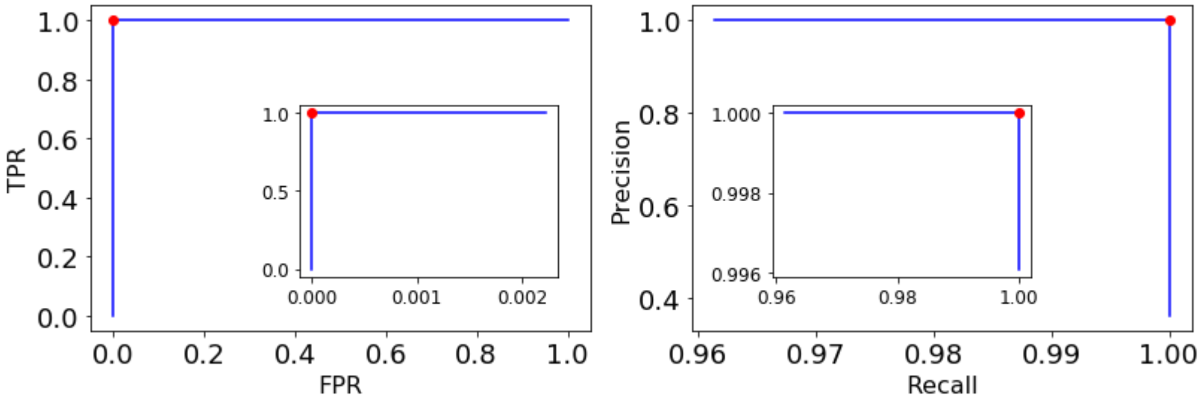

Figure S7 **ROC and precision-recall curve for the anatomy vs background voxel classification of the OPT dataset.** The 0.7 threshold value is represented in red. The figure has been generated via Python 3.7.3 and Microsoft PowerPoint 16.53.

The confusion matrix for the anatomy vs background classification at a threshold of 0.7, which provides a JI value of 100%, is given by the Table S5

	Predicted: Negative	Predicted: Positive
Actual: Negative	18844 (TN)	0 (FP)
Actual: Positive	0 (FN)	10723 (TP)

Table S5 Confusion matrix corresponding to a threshold of 0.7 associated to the anatomy vs background classification in the OPT dataset

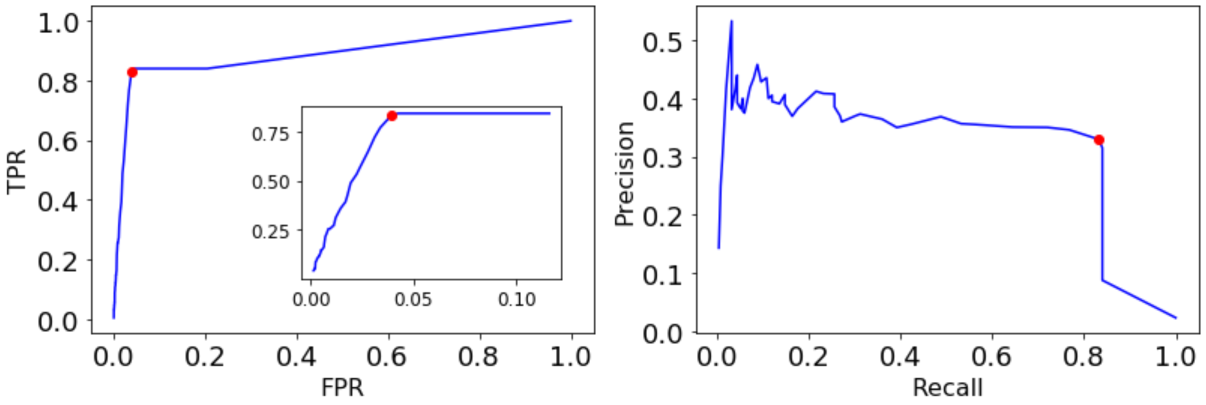

Figure S8 **ROC and precision-recall curve for the plaques vs anatomy voxel classification of the OPT dataset.** The 0.5 threshold value is represented in red. The figure has been generated via Python 3.7.3 and Microsoft PowerPoint 16.53.

The confusion matrix for the plaques vs anatomy classification at a threshold of 0.5, which provides a JI value of 31%, is given by Table S6

	Predicted: Negative	Predicted: Positive
Actual: Negative	10300 (TN)	423 (FP)
Actual: Positive	42 (FN)	208 (TP)

Table S6 Confusion matrix corresponding to a threshold of 0.7 associated to the plaques vs anatomy classification in the OPT dataset

Impact of the DP compression on resolution parameters in PC microscopy

Resolution parameters in a standard PC microscope, such as the Point Spread Function (PSF) and the Modulation Transfer Function (MTF), are measured to compare the predictive uncertainty due to raw data noise of the tested segmentation models with that originated from physical resolution uncertainties. Moreover, we estimate resolution parameters from the raw and the DP compressed datasets to show that their values are preserved upon DP compression.

We compare the value of the PSF of an optical microscope with 20x objective that is extracted from the raw and the DP images of 500 nm diameter microspheres. The FWHM of a single microsphere spatial profile, estimated over 10 different spheres, was measured as (882 ± 36) nm and (883 ± 36) nm from the raw and the DP images, respectively (Figure S9a), showing an excellent agreement between the results obtained with the two datasets. By deconvolving the measured profiles with a gaussian function of 500 nm FWHM, we obtain from both raw and DP compressed images a PSF with a 765 nm FWHM, corresponding to 4.5 pixels. Notice that the spread of the synthetic raw distribution shown in **Erreur ! Source du renvoi introuvable.**c, corresponding to the predictive uncertainty associated to the intrinsic noise of the raw image, is around 1 pixel, which is definitely smaller than that provided by the Point Spread Function (PSF) of the microscope.

The MTF, which indicates the image contrast of a microscope,[2,3] was measured for an optical microscope with 5x objective in bright field with the help of a 1951 USAF target (Figure S9b). For each line series, the modulation is calculated as $M = \frac{I_{max}-I_{min}}{I_{max}+I_{min}}$, where I_{max} (or I_{min}) is the maximum (or minimum) value of the averaged intensity profile of the lines (inset of **Erreur ! Source du renvoi introuvable.**a). The value of M is calculated and averaged over 50 raw and corresponding DP images. The decrease of M with the spatial frequency obtained from raw and DP images is shown in **Erreur ! Source du renvoi introuvable.**a, together with quadratic fitting lines. We obtain a cut-off frequency of f_C = (285 ± 26) line pairs mm^{-1} for the raw data and f_C = (284 ± 25) line pairs mm^{-1} for the compressed ones, confirming again an excellent agreement between the two datasets.

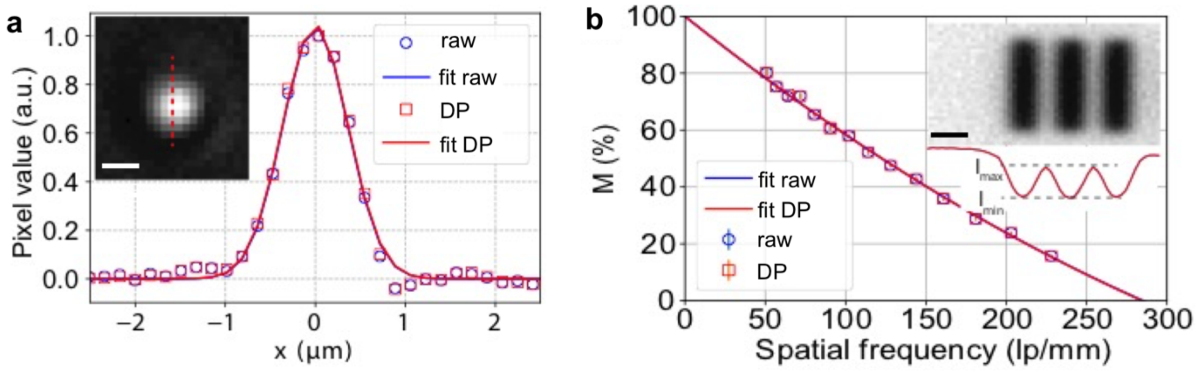

Figure S9 **Resolution parameters in PC microscopy obtained from raw data and after DP compression**

a PSF obtained from the transversal profile of a polystyrene microsphere, imaged with a 20x objective (inset). Gaussian fits of the raw and DP data are used to estimate the FWHM. **b** Modulation transfer function estimated on 50 raw and on corresponding DP files as a function of line pairs mm^{-1}. The cut-off spatial frequency from raw and DP data is estimated with a polynomial fitting. Inset: a typical line series of a 1951 USAF target, from which the contrast of the averaged pixel intensity is measured. The figure has been generated via Python 3.7.3 and Microsoft PowerPoint 16.53.

Impact of the DP compression on resolution parameters in light-sheet microscopy

For the same reasons previously discussed, we measure the PSF of the LS microscope from a 3D stack of 796 images of 100 nm diameter microspheres. In particular, we estimate the PSF from 10 microspheres selected in the raw dataset, as well as in the DP compressed one. As we are interested in comparison of the resolution parameters estimates from the raw and the DP compressed images, we did not implement the pre-processing pipeline utilized in [4] and do not exclude the possibility of beads aggregates.

The lateral PSF, obtained from the average of the FWHM of the x and y spatial profiles (Figure S10a and b), turned out to be (15.4 ± 5.1) µm and (15.3 ± 5.2) µm for the raw and the DP images, respectively. The axial PSF, i.e. the FWHM of the z profile (**Erreur ! Source du renvoi introuvable.**e), is (20.1 ± 6.5) µm and (20.2 ± 6.7) µm for the raw and the DP images, respectively. We conclude that the values from the two datasets, as well as their statistical dispersion, are in very good agreement. Moreover, also in the 3D case, the prediction spread associated to the noise of the raw data, as shown in 3g is around 2-3 voxels, is definitely smaller than that provided by the Point Spread Function (PSF) of the microscope.

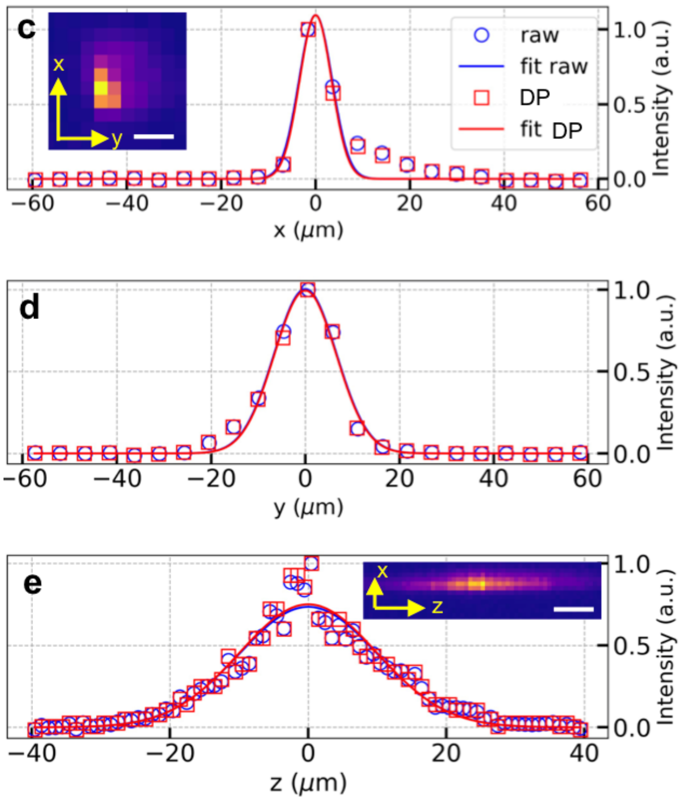

Figure S10 **Measurement of the PSF in light-sheet microscopy obtained from raw data and after DP compression**

Lateral and axial PSF obtained from the spatial profile along x (a), y (b) and z direction (c) of a single 100 nm polystyrene microsphere imaged (insets) with a 1x objective. The figure has been generated via Python 3.7.3 and Microsoft PowerPoint 16.53.

References

1. *EMVA Standard 1288, Standard for Characterization of Image Sensors and Cameras. Release* (2016).

2. Goodman, J. W. Introduction to Fourier Optics 3ed. *Roberts & Company Publishers* (2005).

3. Boreman, G. D. *Modulation Transfer Function in Optical and Electro-Optical Systems. Modulation Transfer Function in Optical and Electro-Optical Systems* (SPIE, 2001).

4. Voigt, F. F. *et al.* The mesoSPIM initiative: open-source light-sheet microscopes for imaging cleared tissue. *Nat. Methods* **16**, 1105–1108 (2019).
